# PK/PD integration of florfenicol alone and in combination with doxycycline against *Riemerella anatipestifer*

**DOI:** 10.3389/fvets.2022.975673

**Published:** 2022-09-08

**Authors:** Huilin Zhang, Yingxin Huang, Jiao Yu, Xujing Liu, Huanzhong Ding

**Affiliations:** Guangdong Key Laboratory for Veterinary Drug Development and Safety Evaluation, College of Veterinary Medicine, South China Agricultural University, Guangzhou, China

**Keywords:** florfenicol, doxycycline, combination therapy, *Riemerella anatipestifer*, PK/PD model

## Abstract

*Riemerella anatipestifer* (RA) is an important pathogen found in poultry. RA infection can kill ducks and lead to significant economic losses. Seven RA strains with different susceptibility phenotypes were chosen to study the pharmacokinetic/pharmacodynamic (PK/PD) integration of florfenicol (FF) alone and in combination with doxycycline (DOX). The checkerboard assay indicated that synergy [fractional inhibitory concentration index (FICI) ≤ 0.5] was detected in the CVCC3952 strain of RA and that additivity (FICI >0.5 to ≤ 1) was observed in other strains. Static time–kill curves showed that the bactericidal effect of FF against RA was produced at a FF concentration ≥4 MIC, and the antibacterial activity of FF against RA was enhanced from the aspects of efficacy and efficacy in combination with DOX. Dynamic time–kill curves indicated that FF elicited bactericidal activity against the CVCC3857 strain with a reduction ≥4.88 log_10_CFU/ml when the dose was ≥8 mg/L. However, a bactericidal effect was not achieved at the maximum administered dose of FF monotherapy (20 mg/L) for isolates with a MIC ≥4 μg/ml. The effect of FF against RA was enhanced upon combination with DOX. The combination of FF with DOX reduced the bacterial burden ≥4.53 log_10_CFU/ml for all strains with a MIC ≥4 μg/ml. Data were fitted to a sigmoidal E_max_ model. The PK/PD parameters of AUC_24h_/MIC (the area under the concentration–time curve over 24 h divided by the MIC) and %T >MIC (the cumulative percentage of time over a 24-h period at which the concentration exceeded the MIC) of FF for eliciting a reduction of 3 log_10_CFU/ml was 40.10 h and 58.71, respectively. For strains with a MIC ≤ 16 μg/ml, the magnitude of the AUC_24h_/MIC and C_max_/MIC required for a 3 log_10_CFU/ml of bacterial killing was 34.84 h and 4.74 in the presence of DOX at 0.5 MIC, respectively. These data suggest that combination of FF with DOX enhanced the activity against RA strains with various susceptibilities to FF and DOX.

## Introduction

*Riemerella anatipestifer* (RA) infects ducks, geese, turkeys, and other types of poultry. RA infection occurs in goslings and ducklings aged 2–8 weeks, and is characterized by septicemia and infectious serositis (1). The gross lesions of necropsy include fibrinous pericarditis, perihepatitis, airsacculitis, catarrhal enteritis, and neurological symptoms (2). RA infection can kill ducks and lead to significant economic losses. More than 21 serotypes of RA have been identified (3). Use of a vaccine alone has limited effects on the high genetic diversity and serotype variation of bacterial strains, so use of pharmacological agents is an important method to control RA. Animal experiments have shown that ceftiofur, cefuroxime, and enrofloxacin are efficacious agents against RA infection (4–6).

Overuse of antimicrobial agents has led to the development of bacterial resistance. RA is resistant to cephalosporins, aminoglycosides, tetracyclines, and fluoroquinolones (1, 7, 8). Gyuris and colleagues showed that 97.9% of RA strains isolated in Hungary were susceptible to florfenicol (FF). However, there are remarkable differences in resistance depending on the use of antibiotics on different farms. The resistance of RA to FF increased from 11 to 43% from 2006 to 2009 in Taiwan (9). Also, 55.26% of RA isolates collected between 2016 and 2020 were FF-positive to the FF-resistant gene *floR*, and showed a phenotype of resistance or low susceptibility to FF (10). The increasing resistance of RA to common antibiotics can compromise treatment. Studies have indicated that combined use of doxycycline (DOX) and FF has synergistic or additive interactions against *Actinobacillus pleuropneumoniae* and *Pasteurella multocida* (11). The combination of DOX and FF has a partial synergistic action against resistant *Escherichia coli* isolates (12).

FF is a structural analog of thiamphenicol, and is approved for veterinary use only. FF binds irreversibly to the 50S ribosomal subunit of a bacterium, which leads to inhibition of peptidyl transferase activity and prevents subsequent protein formation. FF has stronger antibacterial activity than chloramphenicol. Substitution of the hydroxyl group at C-3 with fluorine enables FF to overcome chloramphenicol-resistant bacteria expressing chloramphenicol acetyl transferases. FF has been approved by the US Food and Drug Administration for treatment of respiratory diseases in cattle and pigs. FF is also used to treat infections caused by *E. coli, Salmonella* species, and *Pasteurella* species in chickens in China (12). FF possesses a long elimination half-life (t_1/2β_) in calves (27.54 h) and pigs (14.46 h), and has a relatively short t_1/2β_ in chickens (6.01 h), geese (2.91 h), and ducks (445 min) (13–17).

DOX is a second-generation tetracycline with broad-spectrum antibacterial activity. It binds to the decoding center of the 30S small ribosomal subunit of microorganisms and inhibits protein synthesis. DOX has been approved by the European Medicines Agency for the prevention and treatment of respiratory and gastrointestinal infections in poultry. Studies on the pharmacokinetics (PK) of DOX in pigs, calves, goats, sheep, chickens, geese, and ducks have been plentiful. DOX possesses good tissue penetration, good oral bioavailability, and a long half-life in the serum of animals. The t_1/2β_ of DOX has been reported to be 21.21 h in 6-week-old healthy Muscovy ducks following intravenous administration (18).

Pharmacokinetic/pharmacodynamic (PK/PD) modeling is an important tool for optimizing the dosage schedule to achieve clinical cure and minimizing the emergence of drug resistance (19). Furthermore, the PK/PD relationship is considered important for development of new antimicrobial compounds by the US Food and Drug Administration and European Medicines Agency (20). Limited information is available on the PK/PD interactions of antibacterial agents against RA.

We undertook four main tasks in the present study. First, the minimum inhibitory concentration **(**MIC) of FF and DOX against RA was determined. Second, the fractional inhibitory concentration index (FICI) was calculated to measure the effects of a combination of antimicrobial drugs using the checkerboard assay. Third, static time–kill curves in a defined artificial medium were established, and the relationship between the kill rate and drug concentration was fitted to the maximum value of the kill rate (E_max_) model. Fourth, the relationship between PK/PD indices and the effect of FF alone and in combination with DOX against RA was investigated in an *in vitro* dynamic PK/PD model. Our study is important for dosing guidance for treatment of RA infections.

## Materials and methods

### Materials

The RA strains of CVCC3952 and CVCC3857 were obtained from the Chinese Veterinary Microorganism Culture Collection Center (Beijing, China). Clinical isolates of RA were supplied by the Laboratory of Veterinary Pharmacology within South China Agricultural University (Guangzhou, China). The isolates were obtained originally from the brains of sick ducks and dead ducks in Guangdong Province (China), and identified by polymerase chain reaction targeting the 16S rRNA gene with Sanger sequencing and matrix-assisted laser desorption ionization time-of-flight mass spectrometry. FF (purity ≥98%) and DOX (purity ≥98%) were purchased from Aladdin Industrial Corporation (Ontario, CA, USA). Tryptic soy broth and tryptic soy agar were purchased from Guangdong Huankai Microbial Technology (Guangzhou City, China). Newborn bovine serum was provided by Guangzhou Ruite Biotechnology (Guangzhou, China).

### MIC determination

The MIC of FF and DOX for RA strains was determined using a broth-microdilution method with Mueller–Hinton broth supplemented with 5% calf serum (21). A series of concentrations of FF and DOX was prepared by twofold dilution with media. To each of the first-to-ninth column of a 96-well plate (Costar 3599; Corning, Corning, NY, USA) was added 100 μl of drug of different concentrations. Then, 100 μl of an exponential-phase culture of RA diluted with medium was added to ensure the final inoculum size was 10^5^ CFU/ml. A growth control (RA inoculum in the absence of antimicrobial agents) and a sterility control were included. The *E. coli* ATCC 25922 strain was used as the quality control. The plate was capped and allowed to incubate for 18 h at 37°C. Then, the absorbance was measured using a spectrophotometer (NanoDrop 2000; Thermo Fisher Scientific, San Jose, CA, USA). The MIC measurement for each drug was repeated thrice.

### Checkerboard assay

The interactions between FF and DOX were evaluated by the checkerboard assay. The concentration range of each antibiotic was from four-fold to one/16-fold of the MIC. The final concentration of bacteria in each well was 5 × 10^5^ CFU/ml. Turbidity was checked after 18 h of incubation at 37°C. The FICI was calculated using the following formula:


$$\begin{array}{l} FICI\;=\;\frac{MIC\;of\;FF\;in\;combination\;with\;DOX}{MIC\;of\;FF\;alone}\\ \;+\;\frac{MIC\;of\;DOX\;in\;combination\;with\;FF}{MIC\;of\;DOX\;alone} \end{array}$$

The FICI was interpreted as follows: FICI ≤ 0.5 denotes “synergy”; FICI >0.5 to ≤ 1 denotes “additivity”; FICI >1 to ≤ 4 denotes “no interaction”; FICI >4 denotes “antagonism.” For each strain, the checkerboard assay was repeated thrice (22).

### Exposure to a static concentration of antibiotic

Four milliliters of blank medium, 0.5 ml of 10-times the final drug concentration, and 0.5 ml of logarithmic-phase RA were added to a bottle in turn and then mixed. Tubes containing 5-ml cultures were exposed to FF alone (0, 0.25, 0.5, 1, 2, 4, 6, 8, 16, and 32-times the MIC) or FF + DOX (0, 0.25, 0.5, 1, 2, 4, 6, 8, 16, and 32 times the MIC concentration of FF in combination with DOX at 0.5 MIC, respectively). Cultures were incubated at 37°C with shaking. Then, aliquots were taken from each bottle at 0, 3, 6, 9, 12, and 24 h for colony counts. Each sample was serially diluted 10-fold. Then, 20-μl dilutions were transferred to agar plates and incubation allowed for 24 h. The limit of detection was 50 CFU/ml. Bactericidal activity was defined as >3-log_10_ reduction in CFU/ml. Bacteriostatic activity was defined as >2-log_10_ reduction in CFU/ml (23).

The kill rate can reflect the efficiency of an antibacterial drug against a pathogen. In the present study, because the maximum antibacterial effect had been reached in 9 h at high concentration, the kill rate from 0 to 9 h was selected to fit different drug concentrations. The E_max_ model can be presented as:


$$\begin{array}{l} E\;=E_{\max}\times Ce^{N}/\left(EC_{50}^{N}+C_{e}^{N}\right) \end{array}$$

where E is the kill rate; E_max_ is the maximum value of the kill rate; C_e_ is the drug concentration; EC_50_ is the concentration at which 50% of the maximum kill rate is reached; N is the Hill coefficient (which describes the steepness of the kill rate–concentration curve).

### *In vitro* dynamic PK/PD model

An *in vitro* one-compartment PK/PD model of infection was employed to examine the antimicrobial efficacy of FF alone and in combination with DOX against RA over 24 h, as described by Xiao et al. (24). The model contained a reserve chamber for fresh tryptic soy broth, a central chamber for drug–bacteria interactions, and a waste storage chamber. The central chamber comprised 200 ml of sterile medium. An inverted 15-ml centrifuge tube with a cellulose-ester membrane (pore size = 0.2 μm) covering the top was placed to prevent the loss of bacteria. The flow rate of the peristaltic pump was set at 0.462 ml/min to simulate the PK of FF (t_1/2β_ = 5 h) in duck plasma. According to the maximum concentration of FF in duck plasma (C_max_), six dose groups (2, 4, 8, 12, 16, and 20 mg/L) were designed for FF in the dynamic modeling for each strain. With respect to combination therapy, DOX was administered as a continuous infusion to simulate its long t_1/2β_ in this model. A series of studies was conducted using different doses of FF in combination with DOX at 0.5 MIC to evaluate the PK/PD target of FF in the presence of DOX against RA. Study arms for bacteria alone and control with FF alone or DOX alone were included for each strain.

Before treatment initiation, 10 ml of a log-phase bacterial suspension and 190 ml of culture medium were introduced into the central chamber of the model and allowed to grow for 2 h. Doses of FF were administered into the central compartment, and the time was regarded as the initial time of the experiment. With regard to combination, a dose of DOX was added to the reserve chamber and central chamber to hold a drug concentration of 0.5 MIC in the whole process. We sampled 0.1 ml before administration (0 h) as well as 0.5, 1, 2, 4, 6, 8, 12, and 24 h after administration for bacterial counting, and 1 ml for determination of the drug concentration. Samples were stored at −20°C until analyses.

### Quantification of FF in the medium

The FF concentration in the medium was measured by high-performance liquid chromatography-tandem mass spectrometry using a system from Agilent Technologies (Santa Clara, CA, USA). Separation was achieved on a C_18_ column (150 mm × 2.0 mm, 5 μm; Phenomenex, Torrance, CA, USA). A mixture of ethyl acetate and ammonia (98:2, *v/v*) was used as the extractant. The mobile phase consisted of solution A (water) and solution B (acetonitrile) at a flow rate of 0.25 ml/min. The elution gradient was: 0 min, 10% B; 0–2 min, 65% B; 2–6 min, 65% B; 6–8 min, 10% B; and 13 min, 10% B. The injection volume was 2 μl. A calibration curve was established with six FF concentrations (0.001–0.2 μg/ml). PK parameters were calculated using WinNonlin 6.1 (Pharsight, Mountain View, CA, USA).

### Fitting and analyses of dynamic time–kill curves

The results of dynamic modeling studies were analyzed using the inhibitory sigmoidal maximum effect (E_max_) PD model. The PK/PD parameters of the area under the concentration–time curve over 24 h (AUC_24h_), C_max_, and the cumulative percentage of time over a 24-h period in which the concentration exceeded the MIC (%T >MIC) were calculated according to a one-compartmental analysis using WinNonlin. The inhibitory sigmoidal E_max_ model was:


$$\begin{array}{l} E=E_{\max}-\left(E_{\max}\;-E_{0}\right)\;\times \;Ce^{N}\;/\;\left(EC_{50}^{N}+C_{e}^{N}\right) \end{array}$$

where E is the antibacterial effect; E_max_ is the change in the control group (log_10_ CFU/ml) from 0 h to 24 h; E_0_ is the maximum value of the antibacterial effect; C_e_ is the value of a PK/PD index (C_max_/MIC, AUC_24h_/MIC, and %T >MIC); EC_50_ is the corresponding PK/PD index that produces a 50% reduction of the maximum antibacterial effect; N is the Hill coefficient.

## Results

### Susceptibility testing

The results of MIC determination and the checkerboard assay are summarized in Table 1. To evaluate the activity of FF and DOX against RA (including phenotypic resistant strains), the MIC of seven RA strains selected ranged from 0.5 to 16 μg/ml. The MIC of DOX on RA ranged from 0.125 to 4 μg/ml. According to the FICI, a synergistic effect was observed when FF was combined with DOX against the CVCC3952 strain of RA, and an additive effect was noted for other strains. The MIC of FF against RA in the presence of DOX at 0.5 MIC in the checkerboard assay is shown in Table 1.

**Table 1:** Minimum inhibitory concentration (MIC) and fractional inhibitory concentration index (FICI) in *in vitro* susceptibility testing.

**Strain**	**MIC of florfenicol**	**MIC of doxycycline**	**FICI**	**MIC of florfenicol (with doxycycline at 0.5 MIC)**
CVCC3857	1	1	0.625	0.125
CVCC3952	0.5	0.125	0.5	0.06
RA38	4	2	0.75	1
RA11	4	4	0.75	2
RA17	8	2	0.75	2
RA4	8	4	1	4
RA24	16	4	0.625	2

### Static time–kill curves

**Figure 1:**
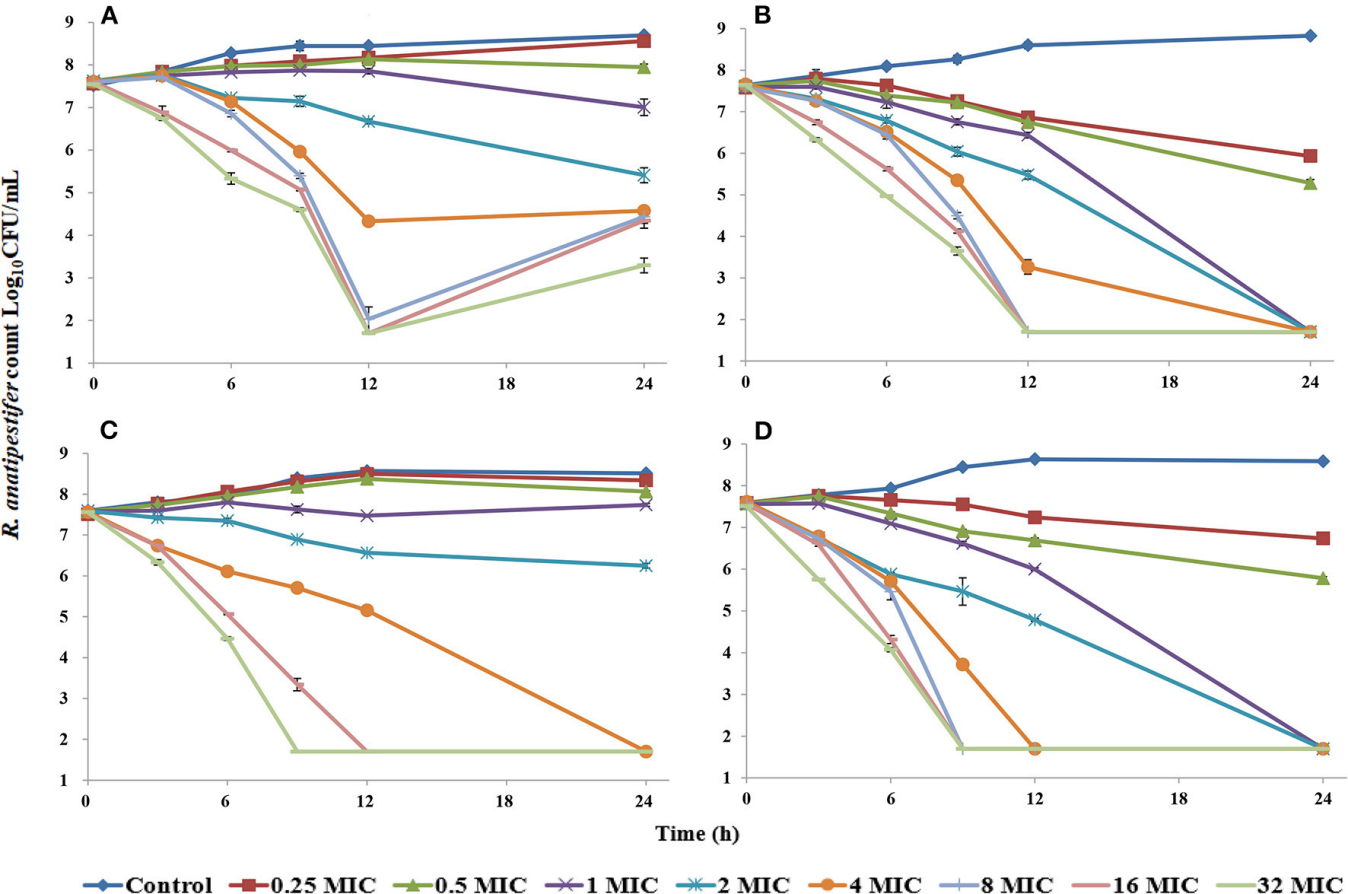
Time–kill studies of florfenicol alone and in combination with doxycycline against *Riemerella anatipestifer* at constant concentrations [FF alone: **(A)**, CVCC3952; **(B)**, CVCC3857; FF in combination with DOX: **(C)**, CVCC3952; **(D)**, CVCC3857].

Florfenicol elicited a reduction of 4.25 log_10_CFU/ml for the CVCC3952 strain and 5.86 log_10_CFU/ml for the CVCC3857 strain of RA at 32 MIC concentration within 24 h (Figure 1). When the FF concentration was <1 MIC, RA showed a slow increase relative to the initial inoculum size. The bactericidal effect of FF against RA was achieved within 24 h when the drug concentration was ≥4 MIC. To determine the impact of DOX on the antibacterial activity of FF, we created static time–kill curves to FF in the presence of DOX. DOX shows activity against RA, so a fixed subinhibitory concentration of 0.5 MIC was selected for DOX. In the presence of DOX at 0.5 MIC, a bacteriostatic effect was observed at FF concentrations <1 MIC, and a bactericidal effect was observed at FF concentrations ≥1 MIC.

**Figure 2:**
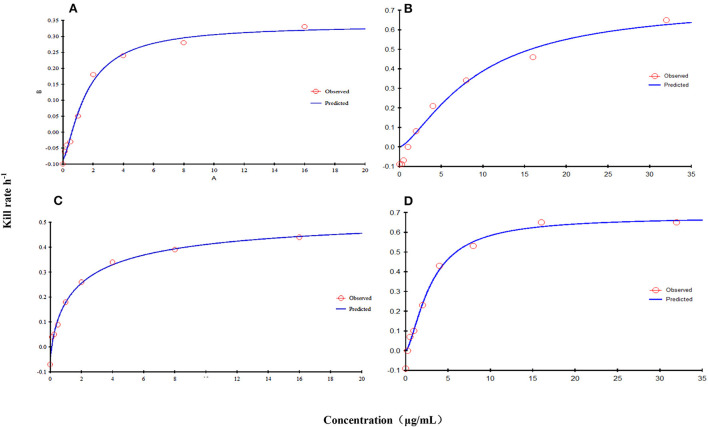
Relationship between the kill rate and concentration obtained from the E_max_ model at 0–9 h [FF alone: **(A)**, CVCC3952; **(B)**, CVCC3857; FF in combination with DOX: **(C)**, CVCC3952; **(D)**, CVCC3857).

The relationship between the kill rate and concentration is presented in Figure 2 (time interval was 0–9 h): the kill rate increased with increasing concentration. The relationship between the concentration and kill rate was fitted by the E_max_ model, and the obtained parameters of E_max_, EC_50_, and the Hill coefficient are presented in Table 2.

**Table 2:** Estimation of the kill-rate parameter derived from the E_max_ model which fitted the data from the *in vitro* static time–kill curve at an interval of 0–9 h.

**Group**	**E_max_ (log_10_ CFU/ml/h)**	**EC_50_ (μg/ml)**	**Hill coefficient**	***R* ^2^**
A	0.34	1.57	1.38	0.996
B	0.73	9.15	1.42	0.987
C	0.54	1.74	0.73	0.997
D	0.68	3.00	1.51	0.993

E_max_, maximum value of the kill rate at an interval of 0–9 h; EC_50_, concentration at which 50% of the maximum kill rate was reached; R^2^, correlation coefficient of the relationship between the experimental value and predicted value.

### PK in the *in vitro* dynamic model

**Figure 3:**
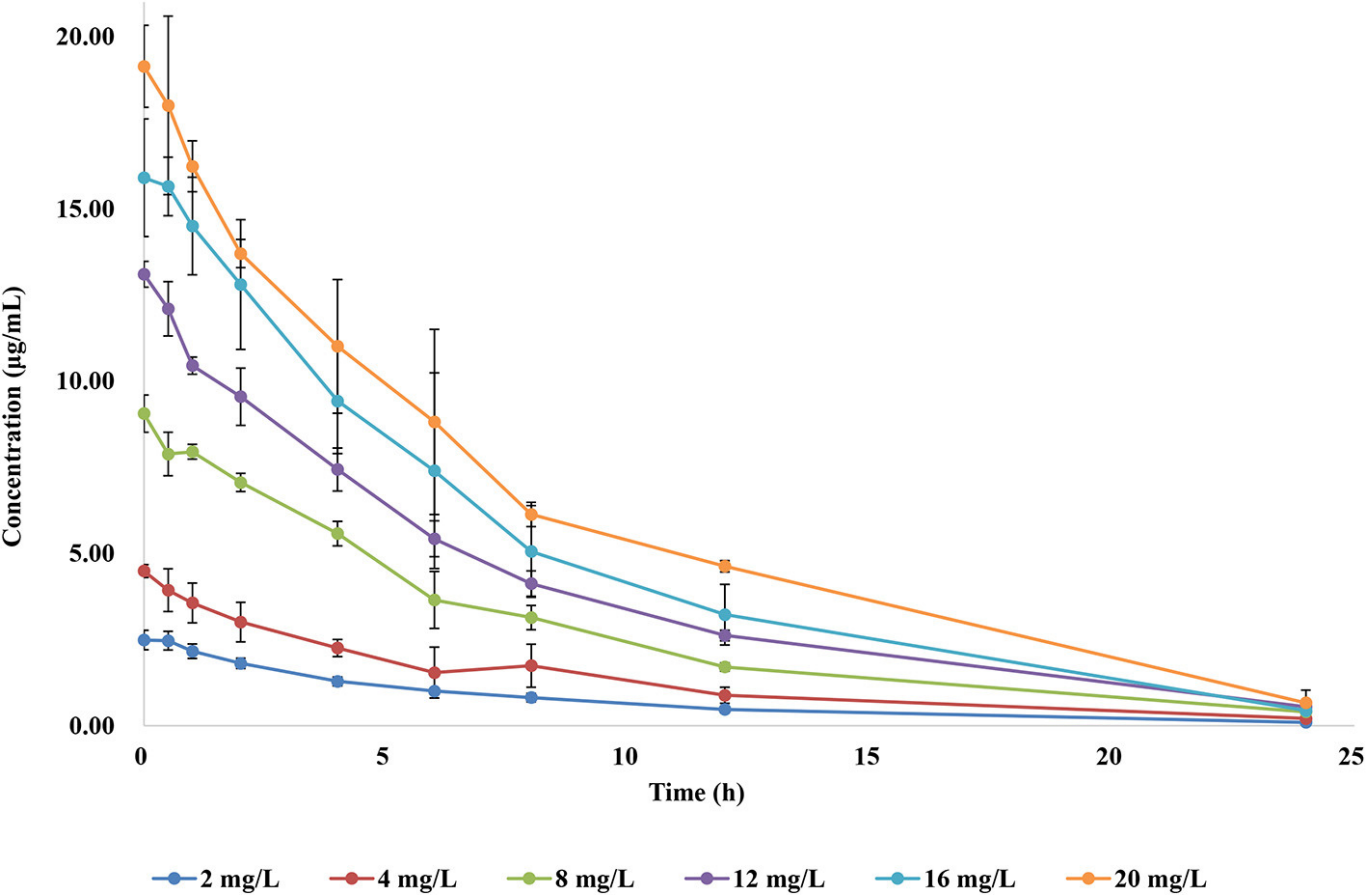
Concentration–time curves of florfenicol in the dynamic model.

To simulate the antibiotic exposure obtained by various treatment doses, six dose groups were designed based on clinical studies. The concentration–time curves of the *in vitro* dynamic model for FF are shown in Figure 3. Data were analyzed according to a one-compartment model by WinNonlin. The half-life was calculated to be 4.995 ± 0.25 h. AUC_24h_ and C_max_ after each drug administration was 17.54–145.56 μg h/ml and 2.52–19.13 μg/ml, respectively.

### PK/PD modeling of FF alone against RA

**Figure 4:**
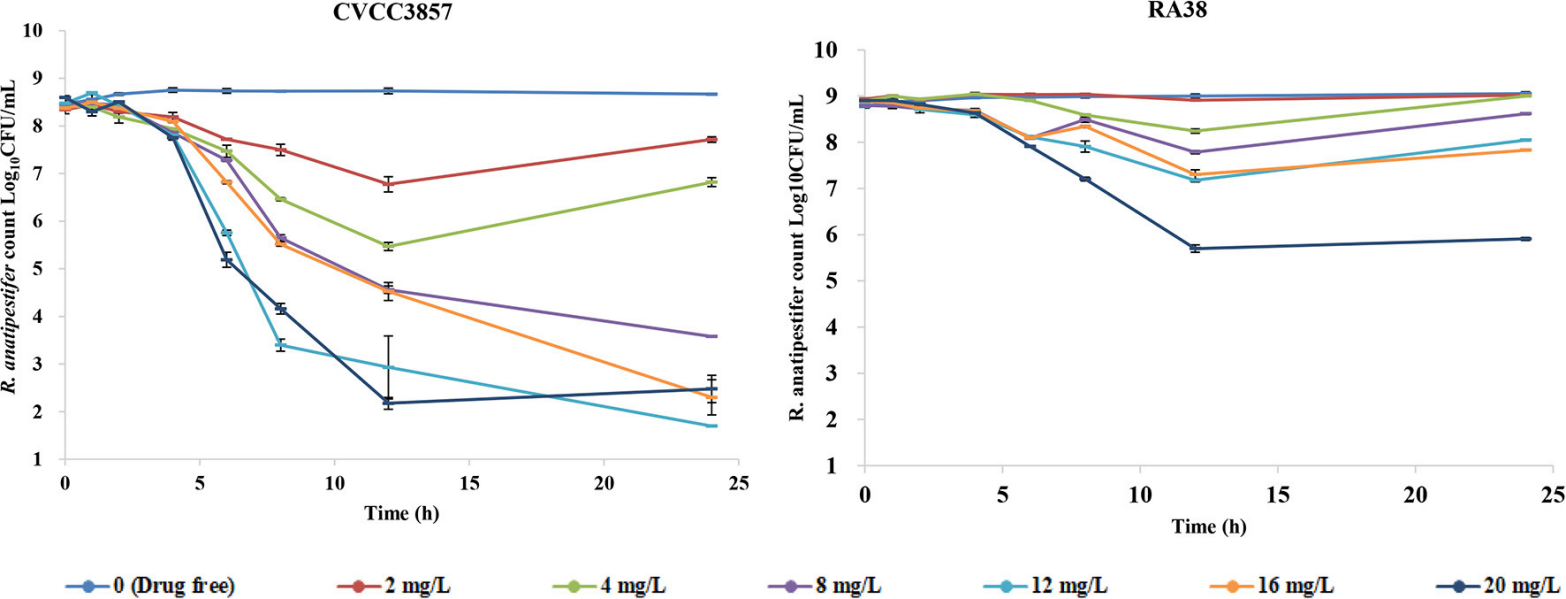
Dynamic time–kill curves of florfenicol alone against *R. anatipestifer*.

Figure 4 shows the antibacterial efficacy of FF alone against RA at six simulated dosing regimens: escalating doses of FF resulted in marked killing of the CVCC3857 strain of RA. The bacterial count decreased by 0.63 log_10_CFU/ml and 1.60 log_10_CFU/ml, respectively, at the FF doses of 2 and 4 mg/L within 24 h. If the dose was ≥8 mg/L, then FF showed bactericidal activity against the CVCC3857 strain of RA, and a reduction of 4.88 log_10_CFU/ml was reached at 8 mg/L within 24 h. However, for the RA38 strain with a MIC of 4 μg/ml for FF, a bactericidal effect was not observed at any dose within 24 h. The maximum reduction was 2.99 log_10_CFU/ml.

**Figure 5:**
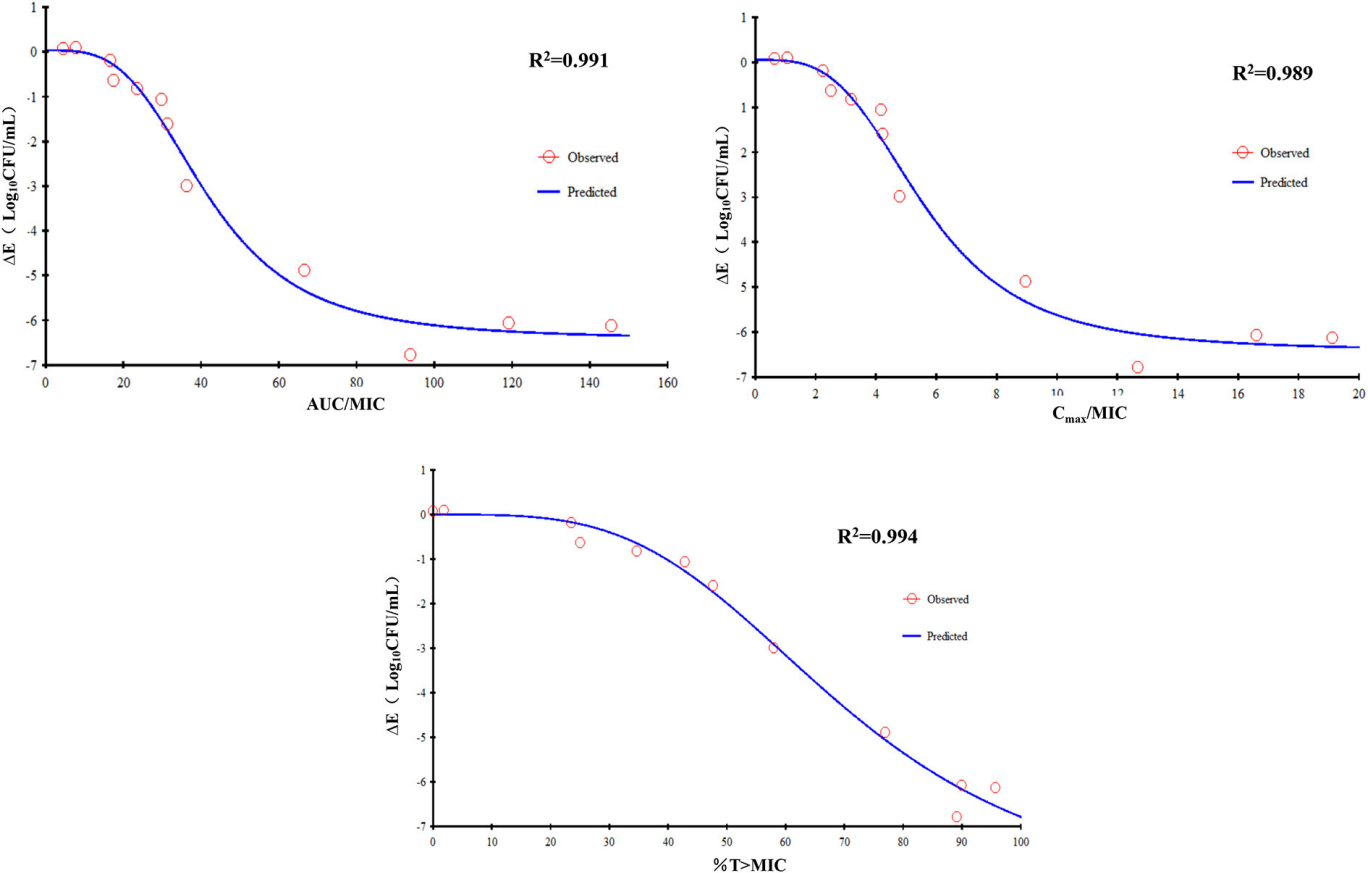
E_max_ relationships for the three PK/PD parameters of florfenicol used alone vs. the antibacterial effect.

The E_max_ relationship of the three PK/PD parameters vs. the antibacterial effect of FF used alone against the CVCC3857 and RA38 strains of RA are shown in Figure 5. The *R*^2^ of AUC_24h_/MIC, C_max_/MIC, and %T >MIC with an antibacterial effect was 0.991, 0.989, and 0.994, respectively. The AUC_24h_/MIC, C_max_/MIC, and %T >MIC for eliciting a reduction in 3 log_10_CFU/ml was 40.10 h, 5.43, and 58.71, respectively.

### PK/PD modeling of FF in combination with DOX against RA

**Figure 6:**
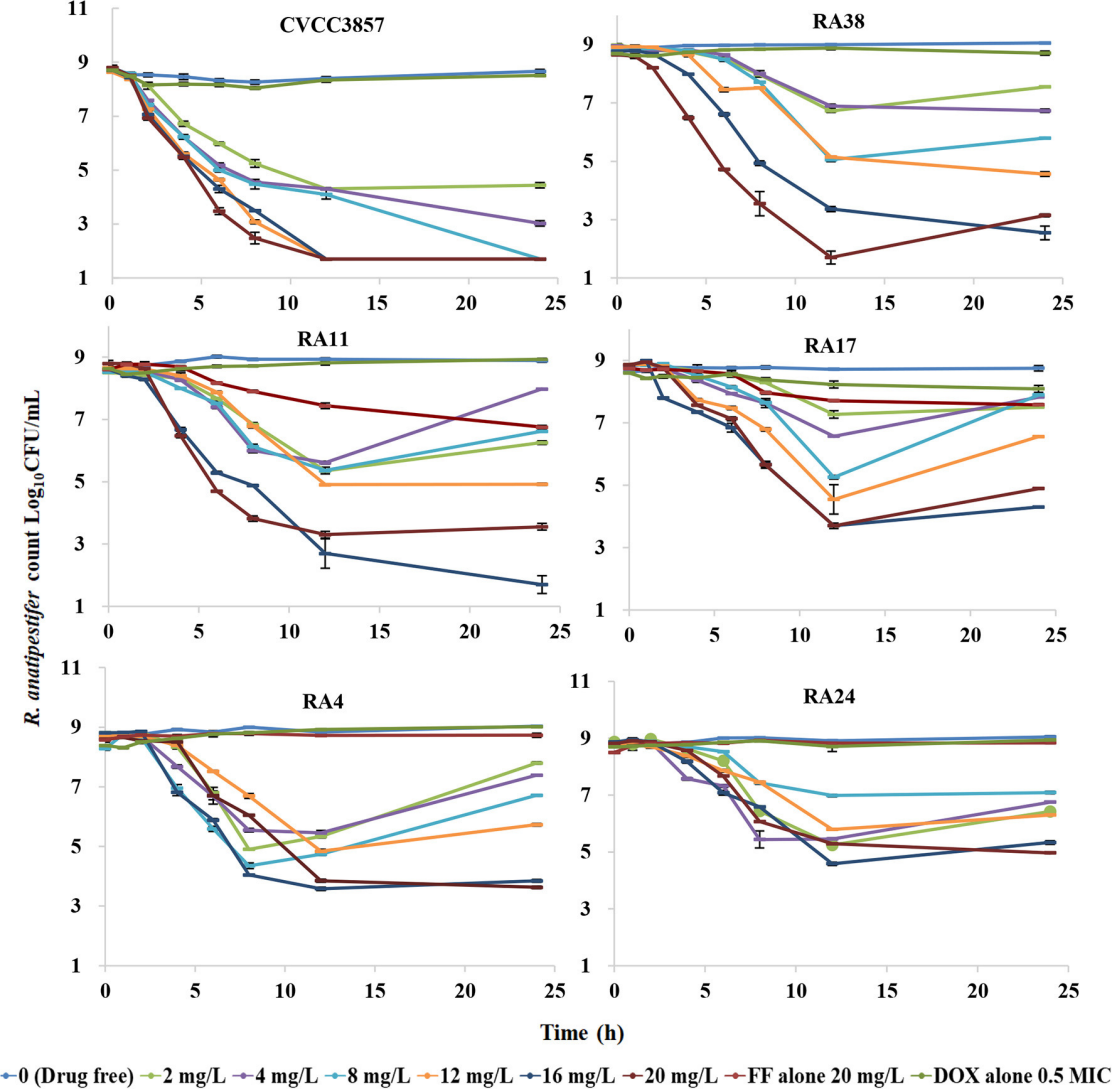
Dynamic time–kill curves of florfenicol in combination with doxycycline against *Riemerella anatipestifer*.

The dynamic time–kill curves of DOX in combination with FF against RA strains are shown in Figure 6. Growth control, the dose of FF alone at 20 mg/L, and DOX alone at a constant concentration of 0.5 MIC were included in all tests. Marked reductions were documented in the presence of DOX at 0.5 MIC within 24 h: a reduction of 4.31 log_10_CFU/ml was produced using FF (2 mg/L) against the CVCC3857 strain. Bactericidal activity was reached at FF ≥8 mg/L against the RA38 strain. For RA11, RA17, RA4, and RA24 strains, the maximum reduction of bacteria (in log_10_CFU/ml) was 6.91, 6.91, 4.53, and 4.97, respectively, at FF ≥16 mg/L.

**Figure 7:**
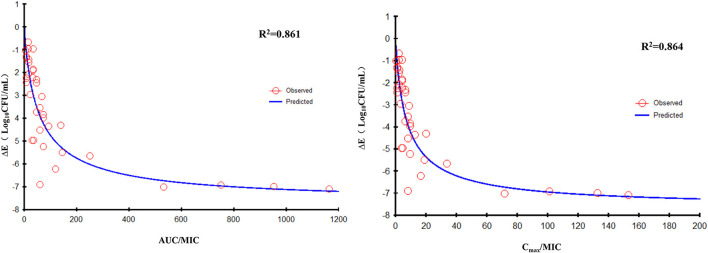
E_max_ relationships for the three PK/PD parameters of florfenicol in combination with doxycycline vs. the antibacterial effect of different isolates.

The data for FF combined with DOX against six RA strains were fitted to the sigmoidal E_max_ model. The PK/PD parameters that correlated most closely to efficacy were AUC_24h_/MIC (*R*^2^ = 0.861) and C_max_/MIC (*R*^2^ = 0.864; Figure 7). The relationship between antibacterial efficacy and PK/PD parameters was assessed by the sigmoidal E_max_ model, and the obtained parameters of E_0_, E_max_, EC_50_, and Hill coefficient are presented in Table 3. The magnitude of AUC_24h_/MIC and C_max_/MIC required for bacterial killing of at 3 log_10_CFU/ml was 34.84 h and 4.74, respectively.

**Table 3:** Estimation of PK/PD parameters derived from the E_max_ model.

**PK/PD parameter**	**E_max_ (log_10_CFU/ml)**	**EC_50_**	**E_0_ (log_10_CFU/ml)**	**Hill's coefficient**	***R* ^2^**	**Three reductions of log_10_CFU/ml**
**Florfenicol alone**					
AUC_24h_/MIC (h)	0.04	41.69	−6.43	3.40	0.991	40.10
C_max_/MIC	0.05	5.62	−6.43	3.38	0.989	5.43
**Florfenicol in combination with doxycycline**					
AUC_24h_/MIC (h)	0	58.78	−7.73	0.87	0.861	34.84
C_max_/MIC	0	7.8	−7.67	0.89	0.864	4.74

## Discussion

Widespread use of FF has led to changes in the susceptibility of RA to FF. The MIC of RA strains (*n* = 66) isolated in Taiwan ranged from ≤ 0.5 to 16 μg/ml, and nine strains possessed the FF-resistant gene *floR* with a MIC ≥4 μg/ml (9). One study indicated the MIC_90_ of RA isolates (*n* = 103) obtained from ducks to be 8 μg/ml during 2008 and 2010 in Southern China (25). The MIC_50_ and MIC_90_ of RA isolates (*n* = 105) from sick ducks or sick geese was 1 and 4 μg/ml, respectively, in Guangdong Province (China), and the MIC of insolates ranged from 0.125 to 16 μg/ml (26). In the present study, the MIC of FF for seven RA strains ranged from 0.5 to 16 μg/ml. The strains selected in our study are important for guiding drug therapy against RA. Similar to other reported pathogens, synergistic and additive effects were observed in the checkerboard assay of FF and DOX against RA. The degree of reduction in the MIC for FF could reach 87.5% (1/8 MIC) upon combination with DOX at 0.5 MIC.

Static time–kill curves demonstrated FF to have a significant effect against RA. At a dose of 30 mg/kg, a C_max_ (in μg/ml) of FF in duck plasma of 18.91, 17.86, and 13.88 has been reported in separate studies (27–29). Considering the FF concentration in animals and the MIC of strains, the CVCC3952 and CVCC3857 strains were selected for the static time–kill test. The concentration series was set to 0–32 MIC. A bactericidal effect was desirable if the FF concentration was 1 MIC combined with a DOX concentration of 0.5 MIC. The kill rate of FF in the presence of DOX at 0 h to 9 h was higher than the kill rate of FF alone deduced from the parameters in Table 2. The antibacterial activity of FF against RA improved from the aspects of efficacy and efficacy upon combination with DOX. A certain degree of regrowth was observed in the monotherapy regimens of FF against the CVCC3952 strain at 12–24 h (Figure 1A). This problem could be overcome by combining FF with DOX (Figure 1C). In addition, the drug-tolerant persistence of RA was eliminated by combining two or three antimicrobial agents (21).

We adopted a one-compartment open model to simulate the PK of ducks to study the effect of dynamic drug concentrations against RA. Due to the rapid absorption of FF following extravascular administration, absorption was not considered in this model. Seven strains with different susceptibility to FF were tested in *in vitro* models to ensure that our results were robust. Although immune factors in animals have not been considered, studies using *in vitro* models have allowed examination of a direct relationship between dosing regimens and bacteriological effects. The *in vitro* dynamic time–kill curve characterized the activity of the drug against bacteria preliminarily. Then, data were fitted to the sigmoidal E_max_ model to obtain specific PK/PD parameters, and the parameter value corresponding to a certain antibacterial effect was calculated by the PK/PD index obtained. Furthermore, PK/PD interactions were analyzed to determine the dependence of the concentration and/or time of the activities (30).

Antibacterial efficacy was characterized in *in vitro* dynamic time–kill curves. If dose ≥8 mg/L, continuous interactions of FF alone resulted in a bactericidal effect for the CVCC3857 strain of RA (MIC = 1 μg/ml; Figure 4A). The values of the PK/PD parameters AUC_24h_/MIC, C_max_/MIC, or %T >MIC are indicators for a drug to achieve an antibacterial effect. An increased MIC due to reduced susceptibility and emergence of resistance affect PK/PD parameters, thereby causing treatment failure (31). For the RA38 strain (MIC = 4 μg/ml), a bactericidal effect was not achieved at the maximum administered dose of FF monotherapy (20 mg/L). Similar results were observed for other RA strains with a MIC ≥4 μg/ml in the present study. The effect of FF against RA was enhanced in the presence of DOX. For the CVCC3857 strain, a reduction of 4.31 log_10_CFU/ml was elicited at a FF dose of 2 mg/L in the presence of DOX compared with a reduction of 0.63 log_10_CFU/ml using FF alone within 24 h. Marked reductions were observed in all strains at different doses of FF administered along with constant infusion of DOX at 0.5 MIC. The combination of FF with DOX reduced the bacterial burden ≥4.53 log_10_CFU/ml from the initial inoculum for the RA38, RA11, RA17, RA4, and RA24 strains with MICs ≥4 μg/ml. Drug combinations delay development of bacterial resistance, enhance antibacterial activity, and treat the infections caused by drug-resistant bacteria.

According to the bactericidal properties of drugs on bacteria, drugs can be divided into time-dependent drugs and concentration-dependent drugs. If the antibiotic concentration is increased, concentration-dependent drugs lead to faster killing, whereas the kill rate of time-dependent drugs might remain constant. In the present study, static time–kill curves showed that a higher concentration of antibiotic elicited a more robust antibacterial effect at 0–9 h. However, at 24 h of the test, bacterial counts dropped to the limit of detection in time–kill studies and dynamic studies if the concentration was >4 MIC or >8 mg/L, respectively. The correlation coefficient of AUC_24__h_/MIC, C_max_/MIC, and %T >MIC with an antibacterial effect was 0.991, 0.989, and 0.994, respectively. Whether the mechanism of action of FF against RA was driven by concentration or time is not known. When FF was combined with DOX, fitting of the E_max_ model showed that the effect on RA was concentration-dependent and driven by AUC_24h_/MIC and C_max_/MIC. The AUC_24h_/MIC, C_max_/MIC, and %T >MIC of FF alone for eliciting a reduction of 3 log_10_CFU/ml was 40.10 h, 5.43, and 58.71, respectively. For strains with a high MIC that could not be treated with FF alone, the value of AUC_24h_/MIC, and C_max_/MIC required to produce a reduction of 3 log_10_CFU/ml was 34.84 h and 4.74 in the presence of DOX at 0.5 MIC, respectively. Hence, combination therapy of FF and DOX with a high MIC was efficacious against the infections caused by RA. Possibly, the presence of DOX reduced the MIC of FF and thereby improved the AUC/MIC ratio.

An appropriate PK/PD index can aid prediction of antibacterial efficacy: %T >MIC for a time-dependent drug or AUC_24h_/MIC for a concentration-dependent drug, but C_max_/MIC is not considered by the European Committee on Antimicrobial Susceptibility Testing (32). Studies have demonstrated that the killing action of FF on the respiratory-tract pathogens of cows and pigs is concentration-dependent and driven by AUC_24h_/MIC. The AUC_24h_/MIC for a reduction of 3 log_10_ has been reported to be 44.02 h for *Streptococcus suis*, 37.3 h for *P. multocida*, and 58.4 h for *A. pleuropneumoniae* in pigs (13, 33), whereas 26.6 h for *M. haemolytica* and 18.1 h for *P. multocida* has been reported for calves (16). The AUC_24h_/MIC for eliciting a reduction of 3 log_10_CFU/ml on RA was 40.10 h in our study. In the studies mentioned above, FF had a long t_1/2β_ in pigs and calves. The AUC_24h_/MIC is the most appropriate index if t_1/2β_ is long. Hence, a single FF dose of 40 mg/kg should elicit a more robust bactericidal effect than a dosing regimen of two doses of 20 mg/kg at an interval of 48 h against the respiratory pathogens in calves (34). The best-fitted PK/PD index can be dependent upon the t_1/2β_ of the drug. It has been shown that the *f* AUC/MIC and *f* T >MIC have a similar predictive capacity for β-lactams, with a preference for *f* T >MIC if t_1/2β_ is relatively short to the dosing interval and *f* AUC/MIC if t_1/2β_ is relatively long to the dosing interval (35, 36). Thus, the selection and magnitude of PK/PD indices differ among animals with a varying capacity to eliminate a drug (20). This is the probably the case for FF with a longer t_1/2β_ sufficient for T >MIC to suppress regrowth of the main population, so AUC_24h_/MIC is the best predictor in pigs and calves. In the present study, the correlation coefficient of AUC_24__h_/MIC and %T >MIC with an antibacterial effect was 0.991 and 0.994, respectively. Also, %T >MIC was as suitable as AUC_24h_/MIC as the PK/PD index for predicting bacterial killing. Alternatively, a shorter interval should be considered in ducks due to the short t_1/2β_ of FF.

## Conclusions

Synergistic and additive effects were observed in the checkerboard assay of FF and DOX against RA. The antibacterial activity of FF against RA was improved from the aspects of efficacy and efficacy in combination with DOX. Due to a short t_1/2β_, the obvious concentration-dependent characteristics of FF against RA were not observed. The parameters of AUC_24h_/MIC, C_max_/MIC, and %T >MIC of FF for eliciting a reduction of 3 log_10_CFU/ml was 40.10 h, 5.43, and 58.71, respectively. The effect of FF against RA was enhanced by combination with DOX; the AUC_24h_/MIC and C_max_/MIC required to elicit a reduction of 3 log_10_CFU/ml was 34.84 h with a MIC ≤ 16 μg/ml for RA strains. These data provide a novel approach for combating RA infections.

## Data availability statement

The original contributions presented in the study are included in the article/supplementary material, further inquiries can be directed to the corresponding author.

## Author contributions

HD and HZ conceived and designed the experiments, revised the manuscript, and supervision of the entire project. HZ, YH, JY, and XL performed the experiments. HZ analyzed data and wrote the manuscript. All authors have read and approved the final manuscript.

## Funding

This work was supported by the National Natural Science Foundation of China (Grant No. 31972733).

## Conflict of interest

The authors declare that the research was conducted in the absence of any commercial or financial relationships that could be construed as a potential conflict of interest.

## Publisher's note

All claims expressed in this article are solely those of the authors and do not necessarily represent those of their affiliated organizations, or those of the publisher, the editors and the reviewers. Any product that may be evaluated in this article, or claim that may be made by its manufacturer, is not guaranteed or endorsed by the publisher.
